# Killing Two Birds with One Stone: Is the COVID-19 Vaccination Campaign an Opportunity to Improve Adherence to Cancer Screening Programmes? The Challenge of a Pilot Project in a Large Local Health Authority in Rome

**DOI:** 10.3390/vaccines11030523

**Published:** 2023-02-23

**Authors:** Alessandra Sinopoli, Valentina Baccolini, Enrico Di Rosa

**Affiliations:** 1 Department of Prevention, Local Health Authority Roma 1, 00193 Rome, Italy; 2 Department of Experimental Medicine, University of Rome “Tor Vergata”, 00133 Rome, Italy; 3 Department of Public Health and Infectious Diseases, Sapienza University of Rome, 00185 Rome, Italy

**Keywords:** prevention, COVID-19 vaccination campaign, healthy behavior

## Abstract

The COVID-19 pandemic has affected health services worldwide. The suspension of cancer screening programs during the lockdown period, coupled with the other measures taken to limit the SARS-CoV-2 spread, contributed to the idea that cancer preventive interventions are deferrable. In this opinion paper, we present some data on cancer screening coverage in one of the largest Local Health Authorities in Italy in recent years. Within this context, we introduce the benefits of a pilot project in which we took advantage of the great attention on the COVID-19 vaccination campaign to improve screening uptake. In this project, we offered men and women eligible for cancer screening the opportunity to book appointments while waiting to be vaccinated. In addition, trained healthcare personnel were available on-site to discuss any barriers to participation with the attendees. Despite the project having only just started, preliminary results are encouraging, with positive feedback from the attendees. In conclusion, we advocate for the need to adopt a comprehensive approach when it comes to population health, and we use this project as an example to discuss how it is possible to contribute to minimizing the long-term impact of the COVID-19 pandemic with resources already in place.

The Coronavirus Disease 2019 (COVID-19) pandemic has deeply affected healthcare delivery across the world [1]. The increase in morbidity and mortality due to SARS-CoV-2 infection caused important consumption of hospital resources [2]. In addition, many countries such as Italy have documented major reductions in healthcare service utilization with greater impacts among people with less severe illnesses [2]. Among others, focusing resources to primarily mitigate the virus spread has determined a dramatic decrease in cancer screening and preventive care activities: elective visits have been rescheduled, non-emergency medical procedures have been canceled or postponed, and cancer screening programs have been suspended for a few weeks during the lockdown period, even though non-homogenously across the country [3]. Furthermore, the enforcement of stay-at-home guidelines coupled with changes in public behavior, such as avoiding attending hospital as much as possible to limit the risk of COVID-19 infection, contributed to the perception of preventive activities as something that could be deferrable, therefore limiting the effectiveness of routine screening by reducing the benefits of early detection [4].

Unfortunately, adherence to cancer screening programs has not yet returned to the pre-pandemic level in many Italian healthcare facilities, despite being a vital component of the Essential Levels of Care (LEA) since their establishment in 2001 [5]. LEAs are the services and benefits that the National Health Service is required to provide to all citizens, free of charge or upon payment of a participation fee (ticket), with the public resources collected through general taxation. Currently, all regions must implement organized screening programs related to the secondary prevention of cervical, breast, and colorectal cancer. They involve active invitation of the target population, free testing and treatment, quality assurance in all stages of the process, and a monitoring system in which each regional program sends data to the National Screening Monitoring Centre [6]. However, despite the efforts made by the Ministry of Health to make oncological screening available to all citizens throughout the country, organized screening programs have suffered from a slow implementation phase since their introduction. Furthermore, they are characterized by geographical differences, with different procedures and programs in place according to the region of residence [7]. Within this context, for early cervical cancer detection, the Lazio Region offers (i) women aged between 25 and 29 years a cervical smear test every three years, and (ii) women aged between 30 and 64 years an HPV test every five years (Figure 1). In addition, women aged between 50 and 74 years are invited to undergo a mammogram test every two years to be screened for breast cancer. Lastly, both women and men between 50 to 74 years are offered a fecal immunochemical test every two years for effective colorectal cancer prevention. The selected method of invitation of the eligible populations is through personal letters directly sent to their address, letters in which all invitees can find the information required to call the cancer screening center located in their Local Health Authority (LHA) of residence and schedule an appointment that best matches their availability. Then, for all three screening programs, negative test results are sent via letters, whereas in the case of positive test results individuals are contacted through phone calls by trained healthcare workers in order to discuss the following healthcare pathway. 

The effectiveness of cancer screening programs is associated with the timely management of patients. This is because timely lesion identification might allow for an early-stage diagnosis that would lead to more conservative treatments and prevent serious complications [8]. For this reason, such effectiveness strongly relies on adherence rate [9]. There are different ways to define and measure screening uptake, but increasing the proportion of individuals who undergo cancer screening tests is a long-lasting challenge [10]. Despite various campaigns and investments, to date, there are still significant differences in participation in all three screening programs across the country, with Northern regions usually achieving higher participation rates than their counterparts. In addition, several determinants that influence attendance to cancer screening programs have been identified, with marked disparities among women in socially or economically disadvantaged groups, as well as in immigrant or ethnic minority populations [11]. Not to mention that the difficulties and delays in organized screening implementation have encouraged the spread of opportunistic screening by private providers, especially in Central and Southern Italy [12], whose long-term effectiveness and cost-effectiveness are still under discussion [13,14,15]. Within this context, the COVID-19 pandemic has tightened up these mechanisms, with variability in recovery pace across Italian regions and programs, but leading to reductions in the participation rates across all types of cancer, with consequent expected delayed diagnoses and increase in the number of avoidable deaths [9].

In the LHA Rome 1, one of the largest in Italy, the coverage of oncological screening programs, defined as the percentage of people screened out of the total eligible population within the specific interval of routine screening, is still below the recommended threshold. In 2022, for instance, invitation letters were sent to 79,300 women for mammography screening, 60,500 women for cytological screening and 173,800 people for fecal blood test screening, an invitation rate in line with the previous years [16]. However, despite always guaranteeing the achievement of the extension of the planned invites (i.e., the percentage of invited people out of the total eligible population), screening uptake has remained consistently unsatisfactory. Indeed, after a slow start-up phase of screening programs that registered population coverage always below the minimum recommended threshold, in 2019 we were able to reach the minimum recommended population coverage for each cancer program (i.e., 25% for colorectal and cervical screening, 35% for mammography screening). The following year, with the advent of the COVID-19 pandemic and the national lockdown imposed on March 9th, 2020, to mitigate the rising infection rate [17,18], the screening services were suspended from March to May 2020 and restarted in June 2020 [19], when all subjects not invited during the suspension period were contacted. Nevertheless, similarly to other centers, LHA Rome 1 experienced a dramatic decline in all cancer screening coverages in 2020, resulting in 13.1% for cervical cancer screening, 11.2% for colorectal cancer screening, and 18.9% for breast cancer screening. Although we observed a slight increase in 2021, the screening coverages remained below the recommended threshold, and this situation was persistent throughout 2022, with low participation rates still limiting the impact of the recovery strategies.

What are the factors associated with low screening adherence rates? Why do participants in previous screening rounds choose not to participate anymore in the screening program? Since the attitude of the eligible population to attend screening invitations seems lower than before the pandemic, new strategies must be found to raise awareness of the importance of preventive testing. Therefore, we thought to take advantage of the great attention of the population regarding COVID-19 [20] and offer the opportunity to participate in the screening tests during COVID-19 vaccination. The COVID-19 vaccination campaign in Italy was a mass immunization campaign that was put in place by the Italian government to respond to the ongoing COVID-19 pandemic [20]. In line with most countries in Europe, in the Lazio region (over 5.7 million residents), as elsewhere in Italy, such a vaccination campaign started on 27 December 2020, with the Comirnaty vaccine being the first vaccine available for administration. Initially, the strategy aimed to rapidly protect frontline healthcare workers, who were the most exposed to the risk of infection, and targeted individuals at high risk of contracting severe COVID-19, such as nursing home patients and elderly people ≥80 years. Then, thanks to rapid vaccine stocking implementation, it was soon possible to extend the offer to other vulnerable groups, and eventually the entire population [21]. All residents in the Lazio region that met the vaccination campaign enrollment criteria and in accordance with the established vaccination schedule by category could be vaccinated anywhere in the region. As of December 2022, LHA Rome 1 serves more than one million inhabitants in its territory with two large COVID-19 vaccination hubs that offer Comirnaty Original/Omicron BA.4–5 vaccination and can be freely accessed with no reservation required. Therefore, we decided to use one of these two vaccination centers of our LHA to start a pilot project. Specifically, since mid-December 2022, we have been offering the population eligible for any of the three cancer screening programs the opportunity to book an appointment while they are waiting to be vaccinated. Moreover, two healthcare workers that were previously trained are in charge of the registration procedures and are available on-site to provide all necessary information on the screening tests, discuss with the attendees the organizational and conceptual barriers to participation and distribute information material on the topic to people requesting it.

Knowledge and awareness are essential for prevention, early detection, and targeted therapy, and represent a key component to ensuring effective treatment. Being aware of the asymptomatic phase of a disease means that people are more likely to take preventive action, including undergoing routine screening tests. Indeed, failure to participate in the screening appointment by responders of the previous screening round indicates that more attention should be given to educational interventions [22,23]. This, coupled with the increase in cancer screening coverage, is the main objective of this project, which is expected to last one year. Preliminary results are encouraging: several appointments to undergo screening tests are made daily, and positive feedback has been registered from the target populations, who have consistently appreciated the opportunity to discuss their doubts about whether to join the screening test with the healthcare personnel as well as be directly informed about the benefits of participating. In this regard, it is well known that communication about cancer screening should be developed and disseminated in ways that empower people to apply information to make decisions about their health, increasing the likelihood that they will adopt interventions of proven effectiveness [24]. Several methods are available to increase adherence to organized screening programs, with results that are different according to a few factors, including the test and the target population of the intervention [25]. However, a key component is information exchange with the healthcare professional to improve knowledge and awareness. In addition, discussing the benefits of oncological services in alternative settings may be helpful in reducing some barriers that usually limit participation in cancer screening programs [26]. Indeed, the scientific literature has already shown how screening uptake is a complex decision-making process influenced by experience, risk perception, culture, and confidence in health authorities [27], which shares the determinants with vaccination intention [28]. Therefore, to engage with the population and promote healthy behaviors, we devised a new method to increase the screening uptake in the current scenario; this strategy aims to enhance the capacity of the healthcare systems and health professionals to customize patient health education and meet the population’s needs. Furthermore, this intervention could improve the ability of participants to communicate with healthcare staff and ultimately increase their capacity to act on health information effectively. Indeed, in addition to national or regional initiatives such as public awareness campaigns, we think it is necessary to work very closely with communities. To this end, people attending COVID-19 vaccination centers represent a large part of the population eligible for the three cancer screening programs, making them a good target for this health intervention.

In conclusion, we believe that health promotion and disease prevention through targeted strategies are critical to improving population health, especially in the context of the COVID-19 pandemic that has disrupted many health services and has had a deep impact on people’s lives. Indeed, we strongly advocate for the need to adopt a comprehensive approach when it comes to health, and this project may represent just an example of how it may be possible to minimize the long-term impact of the COVID-19 pandemic in the current scenario and take advantage of the resources already deployed.

## Figures and Tables

**Figure 1:**
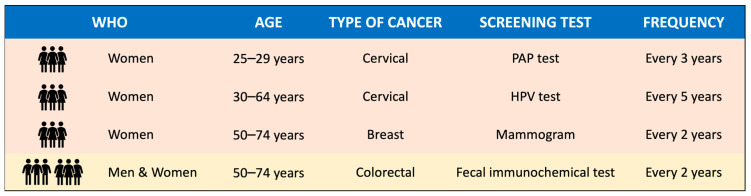
Cancer screening strategies are offered free of charge to all residents of Local Health Authority Rome 1 (Lazio Region, Italy). PAP: Papanicolaou. HPV: Human Papilloma Virus.

## Data Availability

Not applicable.
